# Supplementary material for: The Landscape of Telomere Length and Telomerase in Human Embryos at Blastocyst Stage
Source: Genes (Basel). 2023 May 30;14(6):1200. doi: 10.3390/genes14061200 (PMC10298191; doi:10.3390/genes14061200)
Supplement: Supplementary file 1 [file genes-14-01200-s001.zip › genes-2403241-supplementary.pdf]

Table S1. Telomere length and telomerase gene expression in human blastocysts along with PGT-A diagnosis

**Subject#**	**Embryos**	**Ploidy diagnosis**	**Female age**	**Male age**	**Telomere length**	***TERT mRNA level***
Subject1	Emb#1	47XY, +16	35	29	1.075	553.209
	Emb#2	45XX, -16	35	29	1.362	236.475
Subject2	Emb#3	50XY, +7, +10, +13, +21	44	42	2.723	1005.345
	Emb#4	45XY, -3, +12, -15	44	42	3.712	6581.294
Subject3	Emb#5	46XX, +5, -20	42	40	2.054	791.840
	Emb#6	45XY, -21	42	40	2.730	996.212
Subject4	Emb#7	45XY, -3	41	47	4.486	3024.060
	Emb#8	46XY, +2, -19	41	47	4.234	142.116
	Emb#9	48XX, +2 , +20	41	47	1.466	285.401
Subject5	Emb#10	48XY, +11, +22	44	50	1.897	836.921
	Emb#11	42XO, -8, -15, -20	44	50	2.296	611.990
	Emb#12	46XY, +17, -18	44	50	1.812	280.776
	Emb#13	47XY, +11	44	50	2.256	515.006
	Emb#14	45XXX, -2, -18	45	50	2.366	179.198
	Emb#15	44XX, -1, -13, -17, +18	45	50	1.409	465.602
Subject6	Emb#16	50XY, +2, +3, +5, +8, +10, +11, -14, -21	43	38	2.230	1021.857
	Emb#17	49XY, +2, +18, +22	43	38	1.493	0.193
	Emb#18	46XX, +9, -11	43	38	0.880	213.255
	Emb#19	49XY, +16, +19, +22	44	39	2.303	3107.096
	Emb#20	46XY, +4, -19	44	39	1.311	312.239
	Emb#21	53XY, +4(X3), -8, +11, +12, +19, +20, +21	44	39	2.150	1416.523
Subject7	Emb#22	69XXY	**37**	44	2.074	729.346
	Emb#23	45XX, -15	**37**	44	3.033	2815.888
	Emb#24	46XX, +2, -22	**37**	44	2.052	743.620
Subject8	Emb#25	45XX, -22	40	37	1.020	1400.894
Subject9	Emb#26	48XY, +1, +9q21.11-q21.13, -9, -12, +17, +20, +21	44	27	0.627	2348.987
	Emb#27	45XX, +15, -18, -20	44	27	1.702	218.281
	Emb#28	45XX, -7, +16, -22	44	27	1.421	2159.648
Subject10	Emb#29	45XY, +2, -14, -15, +21, -22	**45**	43	2.025	1392.636
	Emb#30	47XX, -5p13.3-q11.2, -8, +11, +15, +18, -22	**45**	43	1.669	2688.786
	Emb#31	48XX, +19, +20	**45**	43	1.401	875.469
	Emb#32	47XXY, -11, +16, +19, -22	**45**	43	1.568	33.690
Subject11	Emb#33	45XX, -8	41	43	1.124	563.519
	Emb#34	49XYY, +5, +6, +21, -22	41	43	2.052	1165.583
	Emb#35	45XY, +2q22.3-qter, -11, -18pter-p11.23	42	43	2.084	108.411
	Emb#36	46XY, -14, +18	42	43	1.818	251.431
Subject12	Emb#37	46XX, -18, +19	**36**	35	0.880	1550.650
	Emb#38	45XX, -15	**36**	35	0.974	3970.689
	Emb#39	45XX, -3	**36**	35	2.002	4166.658

	Emb#40	45XX, -2pter-p12	**36**	35	1.246	972.976
	Emb#41	47XY, +8	**36**	35	1.130	500.445
Subject13	Emb#42	47XX, +9	40	37	1.339	1205.932
	Emb#43	45XX, -17, +18, -20	40	37	2.188	75.853
	Emb#44	48XX, +19, +22	40	37	1.121	547.299
	Emb#45	46XX, -14, +21	40	37	3.205	666.940
	Emb#46	Complex Abnormal	40	37	1.218	766.369
	Emb#47	48XY, +13, +15	40	37	1.220	334.713
Subject14	Emb#48	45XY, -7pter-7q14.2	23	39	1.612	597.355
	Emb#49	47XY, +2	23	39	0.719	665.343
	Emb#50	***45XX, -10***	23	39	0.973	188.028
	Emb#51	47XX, +7	23	39	0.873	571.146
Subject15	Emb#52	47XX, +1, +14, -21	34	32	1.096	451.165
	Emb#53	44XY, -2pter-p24.1, -8	34	32	1.156	1021.743
	Emb#54	43XY, -2, +4, -5, -10, -15, -18, +21	34	32	1.282	287.770
Subject16	Emb#55	44XY, -11, -16	41	40	1.142	1000.144
	Emb#56	47XY, +22	41	40	1.664	1420.327
	Emb#57	45XY, -11	41	40	1.910	1196.533
	Emb#58	47XY, +20	41	40	1.368	2094.977
Subject17	Emb#59	48XX, +6, +12	40	39	1.339	1143.367
	Emb#60	47XX, -11, +18, +21	40	39	1.646	584.421
	Emb#61	47XY, +15, -18, +19	40	39	1.125	254.187
	Emb#62	45XY, -7, +16, -19	40	39	2.435	681.753
	Emb#63	49XX, +9, +11, +15	40	39	1.375	276.846
	Emb#64	47XY, +16	40	39	1.981	437.249
Subject18	Emb#65	45XX, -21	38	36	2.224	1904.478
	Emb#66	45XY, -2	38	36	1.871	275.114
	Emb#67	45XY, -16	38	36	2.395	124.754
Subject19	Emb#68	Complex Abnormal	37	37	1.793	593.111
Subject20	Emb#69	44XY, -12[mos], +13, -14[mos], -17	43	30	3.077	431.343
	Emb#70	45XY, -12, -21, +22	43	30	5.946	643.781
	Emb#71	47XY, +18q22.2-qter, +22	43	30	2.451	406.369
	Emb#72	51XY, +5, -7p14.1-qter, +9, +10, +14, +16	43	30	2.929	1004.621
	Emb#73	47XX, +14	43	30	2.738	349.990
Subject21	Emb#74	45XX, -15	38	38	6.011	638.846
	Emb#75	47XY, +14	38	38	1.696	226.405
	Emb#76	47XX, -10q25.1-qter, +18	38	38	2.638	186.362
	Emb#77	Complex Abnormal	38	38	1.350	185.202
Subject22	Emb#78	Complex Abnormal	23	32	1.983	236.265
	Emb#79	45XY, -20	23	32	2.752	971.071
	Emb#80	46XY, +18q22.1-qter	23	32	1.931	833.403
	Emb#81	46XY, -16q12.1-qter[mos]	23	32	2.097	393.045

	Emb#82	46XY, -2q14.1-qter[mos]	23	32	2.019	318.888
	Emb#83	45XO, -17q11.2-qter[mos]	23	32	2.083	241.611
Subject23	Emb#84	45XY, -13q31.3-qter, -13pter-q14.3, -19	39	34	1.945	522.407
	Emb#85	47XX, +14, +16, -20	39	34	1.570	723.355
Subject24	Emb#86	47XY, +16	40	40	3.643	177.502
	Emb#87	46XY, +9, -16	40	40	3.125	358.269
	Emb#88	45XY, -11pter-q11[mos], -15	41	40	2.077	1475.982
	Emb#89	45XX, -6q24.2-qter[mos], -13	41	40	1.506	690.041
Subject25	Emb#90	**44XY, +8, -17, -18, -22**	**43**	48	3.241	1.622
	Emb#91	**47XX, +2, +4, -13**	**43**	48	2.706	1838.595
	Emb#92	**46XO, +6**	**43**	48	13.471	317.737
	Emb#93	**46XY, -6, +11, -19, +22**	**43**	48	7.776	366.179
	Emb#94	47XY, +16	**43**	48	1.599	2384.584
	Emb#95	**51XX, +2, +14, +15, +19, +22**	**43**	48	3.639	902.420
	Emb#96	**49XY, +2, +21, +22**	**43**	48	7.446	888.212
	Emb#97	**45XX, -15**	**43**	48	8.031	12505.125
Subject26	Emb#98	46XY	**32**	33	1.243	1003.818
	Emb#99	46XX	**32**	33	1.757	2.644
	Emb#100	**48XX, +2, +3**	**32**	33	1.069	1273.324
	Emb#101	**45XX, -22**	**32**	33	2.848	2593.973
Subject27	Emb#102	47XX, +16	**42**	38	2.810	3002.077
	Emb#103	**45XY, +11, -15, -22**	**42**	38	3.427	523.162
	Emb#104	46XY, +22	**42**	38	1.209	651.713
	Emb#105	**46XY, -7, -8, +18, +22**	**42**	38	1.617	1.892
Subject28	Emb#106	**45XX, -20**	**43**	60	1.627	5527.298
	Emb#107	**47XY, +10, -17, +21**	**43**	60	1.541	2561.415
	Emb#108	46XX, +22	**43**	60	1.189	589.391
	Emb#109	**50XY, +14, +15, +17, +18**	**43**	60	2.071	1092.968
	Emb#110	**45XY, -12, -16, +22**	**43**	60	1.034	35.067
	Emb#111	**45XY, -21**	**43**	60	1.432	1239.359
	Emb#112	**46XY, -7, +15, +19, -20**	**43**	60	1.611	3840.670
Subject29	Emb#113	46XX	39	50	1.000	1.000
	Emb#114	45XY, -13, -19, +22	39	50	1.558	878.699
	Emb#115	45XY, -5, -19, +22[mos]	39	50	1.430	272.808
Subject30	Emb#116	46XX	29	36	1.041	67.364
	Emb#117	47XX, +1[mos], +9[mos], -10	29	36	1.087	4.751
	Emb#118	46XX, +1[mos], -9, -15, +16[mos]	29	36	1.333	220.016
	Emb#119	45XX, -22[mos]	29	36	1.600	185.187

[mos] = mosaic; Complex Abnormal means the details about chromosomal abnormality is not available from PGT-A report.

Table S2. Relative Telomerase Activity in individual embryos with PGT-A diagnosis

Sample	Cq Value	Relative Telomerase Activity by $2^{-\Delta Cq}$	Maternal age	Ploidy Diagnosis by PGT-A
Emb121	23.142	5.763	30	46XX
Emb122	22.872	6.952	33	46XX
Emb123	23.667	4.005	33	47XX, +12[mos]
Emb124	22.861	7.004	39	45XX, -22[mos]
Emb125	23.838	3.557	40	45XY, -11q22.1-qter[mos]
Emb126	22.573	8.549	33	48XX, -7[m], +14[mos], +18[mos], +19 [mos]
Emb127	24.126	2.915	30	45XY, -9pter-p21.1
Emb128	23.695	3.929	34	45XX, -15
Emb129	24.498	2.252	42	45XX, -2
Emb130	24.376	2.450	42	45XX, -22
Emb131	23.360	4.956	42	45XX, -7
Emb132	23.689	3.945	41	47XY,+16
Emb133	23.360	4.956	42	48XY, +16[mos], +21
Emb134	24.270	2.637	42	46XY, -3pter-p24.1, +22
Emb135	23.450	4.655	43	48XY, +3, +22
Emb136	24.407	2.398	43	48XX, +15, +22
Emb137	24.286	2.608	42	48XX, +6, +22
Emb138	26.120	0.731	37	44XX, -9, -10
Emb139	25.617	1.037	42	46XY, +3, -8, +17, -21
Emb140	26.247	0.670	39	46XY, -2, +14, -21, +22
Emb141	27.027	0.390	39	44XY, +1, -2, -15, -19
Emb142	25.555	1.083	39	47XX, +8, +11, -16
Emb143	23.756	3.766	42	45XX, -12, +21, -22
Emb144	24.940	1.658	41	Complex Abnormal
Emb145	23.887	3.438	45	45XX , -9, -17, +21
Emb146	24.430	2.360	42	41XX, -4, -15, -17, -20, -22
Emb147	23.760	3.756	42	43XY, -2, +8, +12, -13, -16, -18[mos], -21[mos]
Emb148	24.960	1.635	43	45XY, -14, -19, +22
Positive Sample	25.669	1.000	NA	NA
Heated Positive Sample	***38.271***	***0.000***	***NA***	***NA***
Water	0.000	0.000	NA	NA

[mos] = mosaic; Complex Abnormal means the details about chromosomal abnormality is not available from PGT-A report.
